# Wounding rapidly alters transcription factor expression, hormonal signaling, and phenolic compound metabolism in harvested sugarbeet roots

**DOI:** 10.3389/fpls.2022.1070247

**Published:** 2023-01-06

**Authors:** Karen K. Fugate, Fernando L. Finger, Abbas M. Lafta, Munevver Dogramaci, Mohamed F. R. Khan

**Affiliations:** ^1^ U.S. Department of Agriculture, Agricultural Research Service (USDA-ARS), Edward T. Schafer Agricultural Research Center, Fargo, ND, United States; ^2^ Departamento de Agronomia, Universidade Federal de Viҫosa, Viҫosa, Brazil; ^3^ Department of Plant Pathology, North Dakota State University, Fargo, ND, United States; ^4^ University of Minnesota Extension Service, St. Paul, MN, United States

**Keywords:** ethylene, jasmonic acid, peroxidase, phenylpropanoid pathway, *Beta vulgaris*

## Abstract

Injuries sustained by sugarbeet (*Beta vulgaris* L.) roots during harvest and postharvest operations seriously reduce the yield of white sugar produced from stored roots. Although wound healing is critically important to reduce losses, knowledge of these processes is limited for this crop as well as for roots in other species. To better understand the metabolic signals and changes that occur in wounded roots, dynamic changes in gene expression were determined by RNA sequencing and the activity of products from key genes identified in this analysis were determined in the 0.25 to 24 h following injury. Nearly five thousand differentially expressed genes that contribute to a wide range of cellular and molecular functions were identified in wounded roots. Highly upregulated genes included transcription factor genes, as well as genes involved in ethylene and jasmonic acid (JA) biosynthesis and signaling and phenolic compound biosynthesis and polymerization. Enzyme activities for key genes in ethylene and phenolic compound biosynthesis and polymerization also increased due to wounding. Results indicate that wounding causes a major reallocation of metabolism in sugarbeet taproots. Although both ethylene and JA are likely involved in triggering wound responses, the greater and more sustained upregulation of ethylene biosynthesis and signaling genes relative to those of JA, suggest a preeminence of ethylene signaling in wounded sugarbeet roots. Changes in gene expression and enzymes involved in phenolic compound metabolism additionally indicate that barriers synthesized to seal off wounds, such as suberin or lignin, are initiated within the first 24 h after injury.

## Introduction

Sugarbeet roots are stored in large outdoor piles or ventilated sheds for up to 250 d prior to processing. However, prior to storage, sugarbeet roots suffer significant injuries from harvest and postharvest operations. Mechanical defoliation and harvesting methods cut, scrape and bruise the root crown and break off the lower tail region of the taproot (Wiltshire and Cobbc, 2000; Bentini et al., 2002). Additional scrapes, breaks, chips and bruises occur from aggressive soil removal practices that agitate roots over chains or rollers, transport methods that drop roots into trucks and dump them at piling stations, and piling operations that drop roots an additional time onto developing storage piles (Campbell and Klotz, 2006). A survey of injury to conventionally harvested and piled roots found that nearly 90% were bruised, 58% had lost the lower portion of the root due to breakage, and 38% were cracked (Steensen, 1996).

Injuries significantly reduce the quantity of white sugar produced from stored roots. Three-fold increases in root respiration, the principal cause of postharvest sucrose loss, have been reported in the days following injury, and elevations in respiration rate that endure for four or more weeks after injury are documented (Wyse and Peterson, 1979; Lafta and Fugate, 2011; Fugate et al., 2016). Breaks in the periderm from wounding facilitate the entry and establishment of storage pathogens, and positive associations between the extent of root injury and the severity of storage diseases are reported (Mumford and Wyse, 1976; Liebe and Varrelmann, 2016; Hoffmann et al., 2018). As disease incidence increases, storage losses escalate since storage pathogens consume sugar, increase the accumulation of non-sucrose carbohydrates such as glucose and fructose that reduce sucrose recovery during processing, and generate heat which accelerates root respiration and storage disease severity within the piles (Campbell and Klotz, 2006; Schnepel and Hoffmann, 2016). Root dehydration also increases with injury, causing additional elevations in root respiration rate and storage rots (Gaskill, 1950; Lafta and Fugate, 2009; Fugate et al., 2016).

Although the extent of root injury and its impact on storage are well documented, knowledge of the molecular events involved in wound-healing in sugarbeet roots is limited. Injured sugarbeet roots are known to synthesize suberin and lignin to seal off wounded sites (Ibrahim et al., 2001; Fugate et al., 2016). The sealing of wound sites occurs over weeks after the injury and is dependent on the temperature at which roots are stored. Injured sugarbeet roots are known to produce ethylene, indicating the potential involvement of this hormone in triggering wound responses (Fugate et al., 2010). The activities of the early glycolytic enzymes, fructokinase, hexokinase and phosphofructokinase, also increase after wounding, presumably to generate the carbon substrates and metabolic energy needed to support elevated respiration rates and biosynthetic processes (Klotz et al., 2006).

While knowledge of wound-healing events is limited in sugarbeet roots, these processes are better characterized in other plant species and organs. The physiological events that seal off wound sites have been investigated in detail in potato tubers, and biochemical changes that generate substrates for barrier-forming reactions have been described in carrot roots (Becerra-Moreno et al., 2015; Lulai et al., 2016). The role of the hormones, jasmonic acid (JA) and ethylene, as signals for wound responses have been described in leaves of many plant species including *Arabidopsis*, tobacco and tomato (Titarenko et al., 1997; Wasternack et al., 2006; Onkokesung et al., 2010). Additionally, wound-induced transcriptomic changes have been described for several plant species and organs including leaves of *Arabidopsis thaliana*, *Lolium temulentum*, cotton, and chickpea, and stems of *Pinus* species (Consales et al., 2012; Chano et al., 2017; Pandey et al., 2017; Dombrowski et al., 2020; Si et al., 2020; Lim et al., 2021). Transcriptional changes due to wounding have also been reported in roots but are limited to a single study which identified 335 wound-induced genes in carrot by suppression subtractive hybridization (Jacobo-Velázquez et al., 2015). The relevance of studies conducted with other plant systems for understanding sugarbeet root wound healing, however, is likely to be limited since the genes, signaling compounds, and even the physiological processes involved in wound healing have been shown to differ between plant species and organs (Rittinger et al., 1987; Saltveit, 2016).

To increase understanding of wound healing in sugarbeet root, research was conducted to determine the effect of wounding on the sugarbeet root transcriptome with respect to time after injury. To achieve this, harvested sugarbeet roots were wounded by agitation in the revolving drum of a pilot scale beet washer to create scrapes, cuts and bruises similar to those sustained by commercially harvested roots. Dynamic changes in gene expression and metabolic responses to wounding were then determined by evaluating roots at varying times after injury. Transcriptional changes were evaluated over the first 24 h after injury, with an initial sample collected 15 minutes after injury since transcriptional, hormonal signaling, and metabolic responses to wounding are known to occur rapidly and be initiated in this time frame in other plant systems (Pandey et al., 2017; Si et al., 2020; Lim et al., 2021). The purpose of this research was to generate new insight into the transcriptional changes and the metabolic signals and pathways that are triggered by wounding in a commercially important crop and a plant organ whose wound-healing processes are largely unknown.

## Materials and methods

### Plant material and treatments

Sugarbeet taproots (variety VDH66156, SESVanderHave, Tienen, Belgium) were grown in a greenhouse in 15 L pots with 16 h days/8 h nights as described by Megguer et al. (2017). After 16 weeks, 48 taproots were harvested, and shoots were removed with a knife. Shoot removal left no petiole material attached to the root but caused a small, flat, transverse wound on the taproot apex. Half of the roots were gently handwashed and these roots served as minimally-wounded, experimental controls. The remainder of the roots were washed in a pilot-scale beet washer (Hallbeck, 1982) for 15 minutes, causing severe wounds in the form of surface abrasions, bruises, and the loss of the root tail. Control and wounded taproots were incubated at 22 °C for 24 h. Tissue samples were collected at 0.25, 2, 4, 8, 12 and 24 h after the wound treatment by removing a transverse section from the middle portion of the tap root. Tissue samples were flash-frozen in liquid nitrogen at the time of collection, lyophilized, and stored at -80°C prior to use. The experiment was conducted as a completely randomized design with four replications, with individual roots as the experimental unit.

### RNA sequencing

Total RNA was extracted from lyophilized tissue (50 mg) using a RNeasy Plant Mini Kit (QIAGEN, Valencia, CA, USA) with an on-column DNase digestion. RNA concentration was determined spectroscopically using a ThermoFisher Scientific NanoDrop ND-1000 (Waltham, MA, USA), and RNA integrity was confirmed by the RIN number generated by an Agilent Technologies 2100 Bioanalyzer (Pal Alto, CA, USA). RNA was fragmented, converted to cDNA using random primers, amplified by PCR, and sequenced by BGI Americas (Cambridge, MA, USA) using a DNBseq platform to generate ≥ 45 M raw reads per sample.

### Enzyme activity assays

Proteins were extracted and assayed for 1-aminocyclopropane-1-carboxylic acid oxidase (ACO) activity following the protocol of Vriezen et al. (1999) with modification. Lyophilized tissue (100 mg) was pulverized in liquid nitrogen. A solution (500 µL) of 300 mM Tris-Cl, pH 7.2, 30 mM sodium ascorbate, and 10% [v/v] glycerol was added to the cold, ground tissue, and the resulting mixture was thawed to a slurry and centrifuged at 18,000*g* for 15 minutes at 4 °C. ACO activity of the supernatant was determined at 30°C in the dark in 5 mL septum-sealed glass vials. Each vial contained a solution (500 µL) of 100 mM Tris-Cl, pH 7.2, 30 mM sodium ascorbate, and 10% [v/v] glycerol, 50 µL of 80 mM 1-aminocyclopropane-1-carboxylic acid (ACC), 40 µL of 3 mM FeSO_4_, and 60 µL of 1 M NaHCO_3_ with all vial contents prewarmed to the reaction temperature. Reactions were initiated by the addition of 100 µL of enzyme extract. After 15 minutes with continuous mixing, ethylene concentration in the vial headspace was measured using a Felix Instruments F-950 Three Gas Analyzer (Camas, WA, USA) in trigger mode. The analyzer was connected to the reaction vial using needle probes inserted through the vial septum and operated at a flow rate of 75 mL minute^-1^.

Phenylalanine ammonia lyase (PAL) activity was assayed according to the protocol of Maldonado et al. (2007) with modification. Finely ground, lyophilized tissue (100 mg) was mixed with of a solution (800 µL) containing 0.1 M sodium borate buffer, pH 8.8, 5 mM 2-mercaptoethanol, 2 mM EDTA and 2% insoluble PVP (w/v), vortexed for 30 s, and centrifuged at 18,000*g* for 30 minutes at 4°C. Activity was assayed at 40 °C by adding enzyme extract (50 µL) to a prewarmed solution (910 µL) containing 50 mM sodium borate buffer (pH 8.8) and 10 mM L-phenylalanine. After 30 minutes with continuous shaking, the reaction was stopped by adding 6 M HCl (40 µL). Product formation was measured by comparing solution absorbance at 290 nm to a standard curve prepared with cinnamic acid solutions of known concentrations.

Peroxidase (POD) activity was determined using the protocol of Fugate et al. (2016) without modification. Total protein concentration of enzyme extracts was determined using Bio-Rad Protein Assay Dye Reagent (Hercules, CA, USDA) and bovine serum albumin as a standard.

### Soluble phenolics

Soluble phenolic compounds were extracted from finely ground, lyophilized tissue and quantified as previously described using gallic acid standards (Fugate et al., 2016).

### Data analysis

RNA-seq data were cleaned using SOAPnuke ver. 1.5.2 (Chen et al., 2018) to remove reads with adapters, reads with >0.1% unknown bases and low-quality reads, leaving >40 M clean reads per sample. Clean reads were mapped to the sugarbeet genome (Dohm et al., 2013) using Bowtie2, ver. 2.2.5 (Langmead and Salzberg, 2012). Gene expression levels were calculated with RSEM ver. 1.2.12 (Li and Dewey, 2011), and differentially expressed genes were detected using DEseq2 (Love et al., 2014). Functional enrichment of DEGs was performed using the phyper function in R. Transcription factors (TFs) were identified by extracting open reading frames (ORFs) from differentially expressed genes using EMBOSS:getorf ver. 6.5.7.0 and aligning ORFs to the TF domains in PlnTFDB ver. 23.0 (Riaño-Pachón et al., 2007) using HMMER ver. 3.0 (Mistry et al., 2013). Heat map and dendrogram of highly expressed transcription factors were generated using Next Generation Clustered Heat Map Tool ver. 2.14.4 (NG-CHM, MD Anderson Cancer Center, Houston, TX, USA). Gene expression heat maps related to ethylene and JA biosynthesis and signaling, and phenylpropanoid pathways were created in OriginPro 2017 (OriginLab Corp, Northhampton, MA, USA). Pearson correlations between enzyme activities and differentially expressed genes were determined using Minitab software (ver. 20.4, State College, PA, USA) with p ≤ 0.05. Significant differences in enzyme activities between wounded and control roots at distinct time points were identified using t-tests with p ≤ 0.05 using Minitab.

## Results

### Quantitative and functional analysis of gene expression alterations

**Figure 1:**
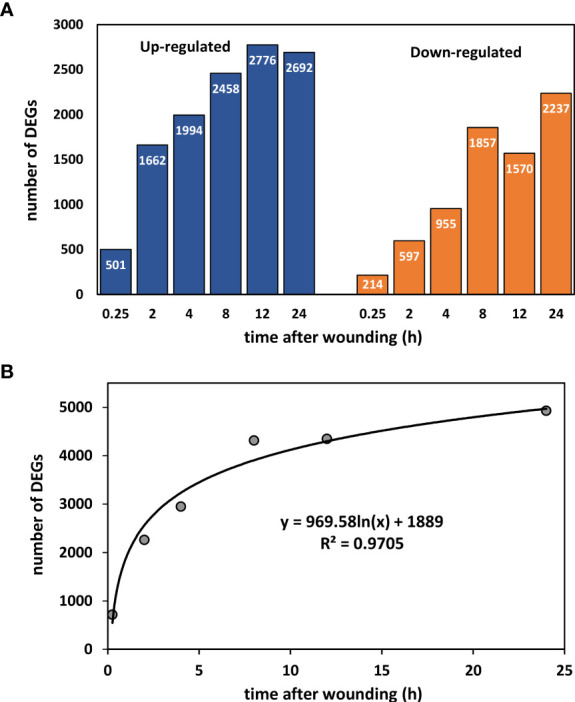
Number of differentially expressed genes (DEGs) between wounded and control roots as a function of time after wounding. **(A)** Up- and down-regulated DEGs after 0.25, 2, 4, 8, 12, and 24 h after wounding. **(B)** Effect of time after wounding on the total number of DEGs.

Wounding resulted in rapid changes in the expression of numerous sugarbeet root genes (**Figure 1**). Within the first 15 minutes, 501 genes were up-regulated and 214 genes were down-regulated in wounded roots relative to controls (**Figure 1A**). The number of differentially expressed genes (DEGs) increased logarithmically during the first 24 h, and after 24 h after injury, a total of 4929 genes were differentially expressed (**Figure 1B**). At all sampling times, more DEGs were up-regulated than down-regulated in wounded roots with the ratio of up-regulated to down-regulated genes greatest at 0.25 to 2 h after wounding. The full list of wound-altered DEGs and their expression with respect to time after wounding is available in **Supplementary Table S1**.

The DEGs induced by wounding participate in a wide variety of biological processes and molecular functions as classified using gene ontology (GO) terms (**Table 1**). The most highly populated GO terms, however, were involved in plant metabolism. Within the biological process gene ontology, the most highly populated GO terms were cellular processes and metabolic processes, which contain genes involved in metabolism at the cellular and organismal level, respectively. Within the molecular function ontology, DEGs involved in metabolism also predominated with binding and catalytic activity as the most highly populated GO terms.

**Table 1:** Classification of differentially expressed genes (DEGs) in wounded roots by biological process and molecular function gene ontology (GO) terms, as a function of time after wounding.

		Number of DEGs					
Ontology	GO term	0.25 h	2 h	4 h	8 h	12 h	24 h
*Biological process*	biological regulation	19	52	82	116	102	112
	cell killing	0	0	0	1	1	1
	cell proliferation	0	0	1	0	0	2
	cellular component organization or biogenesis	8	30	30	55	47	60
	cellular process	44	156	213	316	297	335
	detoxification	0	0	0	0	0	1
	developmental process	6	22	27	31	36	38
	growth	2	5	4	5	7	8
	immune system process	1	3	2	4	4	4
	localization	7	24	33	52	48	70
	metabolic process	41	165	213	310	296	322
	multi-organism process	2	5	8	17	14	14
	multicellular organismal process	6	21	23	29	32	35
	negative regulation of biological process	2	6	10	18	16	20
	nitrogen utilization	1	0	1	1	1	1
	positive regulation of biological process	1	4	0	8	6	6
	regulation of biological process	17	45	69	101	89	94
	reproduction	2	10	10	14	13	18
	reproductive process	2	10	10	14	13	18
	response to stimulus	12	48	68	107	106	108
	rhythmic process	2	1	2	3	3	2
	signaling	5	18	22	32	27	29
*Molecular function*	antioxidant activity	0	7	7	14	13	13
	binding	52	229	281	418	426	472
	catalytic activity	55	222	296	435	443	487
	molecular carrier activity	0	0	2	3	3	2
	molecular function regulator	1	6	11	12	13	15
	molecular transducer activity	0	5	7	6	7	9
	nutrient reservoir activity	0	4	3	9	14	14
	signal transducer activity	1	10	15	14	13	11
	structural molecule activity	1	3	6	6	7	9
	toxin activity	0	0	1	3	1	2
	transcription regulator activity	8	18	23	36	36	33
	transporter activity	8	29	33	58	55	76

**Figure 2:**
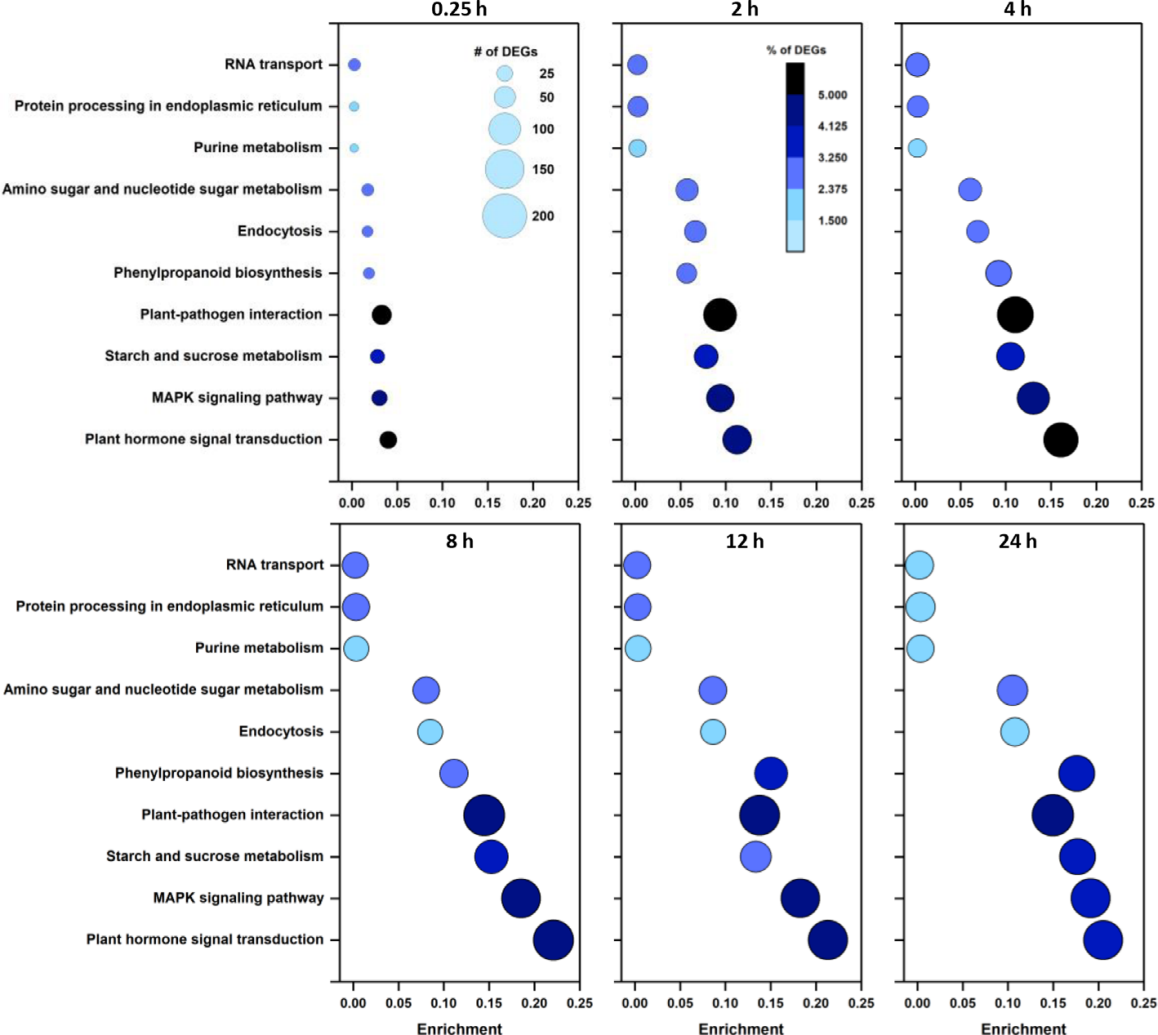
Enrichment of the ten most highly populated KEGG pathways as a function of time after wounding. Size of data points is proportional to the number of DEGs. Color of data points indicates the percentage of pathway genes that are differentially expressed.

Mapping DEGs to KEGG pathways identified enriched pathways and biological functions in wounded roots (**Figure 2**). The most enriched pathway at all sampling times was plant hormone signal transduction. Enrichment of this pathway increased between 0.25 and 8 h and by 8, 12, and 24 h after wounding, more than 20% of plant hormone signal transduction pathway genes were differentially expressed. The MAPK signaling pathway was the second most highly enriched pathway with 19% of the pathway’s genes differentially expressed after 24 h. Among highly enriched KEGG pathways, the greatest number of DEGs belonged to the plant-pathogen interaction pathway. Enrichment of this pathway, however, was lower than the above-mentioned pathways due to the large number of genes assigned to this pathway. Other highly enriched pathways include starch and sucrose metabolism and the phenylpropanoid biosynthesis pathway. Enrichment of the phenylpropanoid biosynthesis pathway lagged behind the enrichment of other highly enriched pathways during the first 8 h after wounding. However, phenylpropanoid biosynthesis pathway enrichment increased in the remaining 16 h of the experiment such that 18% of phenylpropanoid pathway genes were differentially expressed after 24 h.

### Transcription factors and signaling pathways

Wound effects on the expression of signaling-related genes were further analyzed by identifying and evaluating the expression patterns of differentially expressed transcription factors (TFs). During the first 24 h after injury, 514 unique TFs were differentially expressed in wounded roots at one or more sampling time points (**Supplementary Table S2**). The number of DEGs for TFs ranged from 68 to 275 at different sampling times (**Table 2**). The number of TF DEGs generally increased during the first 8 h after wounding and remained relatively unchanged for the next 16 h. At all sampling times, more TF genes were upregulated than downregulated in wounded roots with the ratio of upregulated to downregulated genes greatest at 0.25 and 2 h after wounding.

**Table 2:** Number of differentially expressed transcription factors in wounded sugarbeet taproots as a function of time after wounding.

Time after	Number of transcription factors		
wounding (h)	upregulated	downregulated	total
0.25	54	14	68
2	105	46	151
4	130	88	218
8	159	116	275
12	180	92	272
24	144	115	259

**Figure 3:**
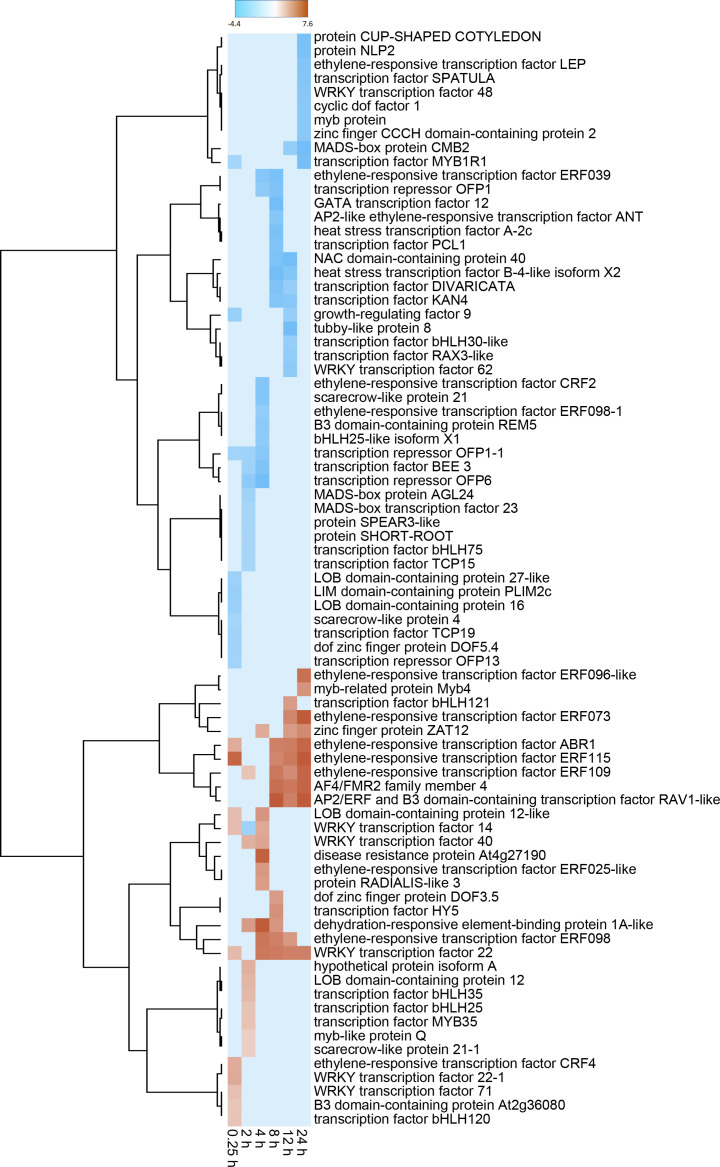
Heat map of changes in transcription factor (TF) gene expression in response to wounding. Differential gene expression is displayed as the log_2_ fold change in gene expression between wounded and control roots at 0.25, 2, 4, 8, 12 and 24 h after wounding for striked out text should be replaced with: the ten most highly up-regulated and down-regulated TF DEGs from each time point. Genes are hierarchically clustered based on their pattern of differential expression over time.

Differentially expressed TF genes belonged to 51 different transcription factor families (**Supplemental Table S3**). The most highly populated TF families were the APETELA2/ethylene-responsive element binding proteins (AP2-EREBP), MYB, basic helix-loop-helix (bHLH), WRKY and NAC families which contained 61, 52, 51, 35 and 20 DEGs, respectively. The identity and expression of the ten most highly elevated and reduced TF genes for each sampling time over the 24 h after wounding are shown in **Figure 3**, with genes grouped by similarity in expression pattern. Within this subset of TF DEGs, members of ethylene-responsive TF families were most abundant, with ten upregulated and five downregulated genes. Moreover, six of these ethylene-responsive genes clustered in a branch of the dendrogram that contained genes that were highly expressed at one or more time points between 8 and 24 h after wounding and were upregulated by more than 100-fold. Also abundant within these TF DEGs were genes belonging to the bHLH (10 genes), MYB (8 genes), and WRKY (8 genes) TF families. The WRKY family DEGS were notable for early but transient upregulation in expression between 0.25 and 8 h, and reduced expression at 12 and 24 h after wounding. Upregulation of WRKY family DEGS, however, was significantly lower than that observed for ethylene-responsive genes.

### Ethylene biosynthesis and signal transduction

**Figure 4:**
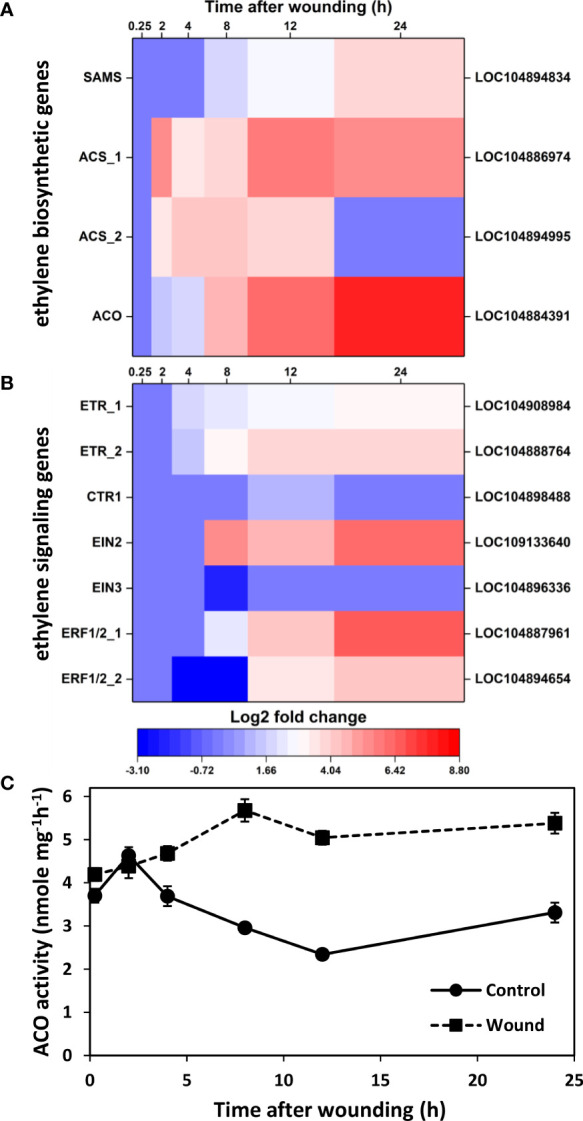
Changes in differential expression with respect to time after wounding for genes involved in ethylene biosynthesis **(A)** and ethylene signaling **(B)** and enzyme activity of 1-aminocyclopropane-1-carboxylic acid oxidase (AOC) **(C)**. Heat maps display the log_2_ fold change in gene expression between wounded and control roots at 0.25, 2, 4, 8, 12 and 24 h after wounding for all pathway genes that exhibited a log_2_ fold change in expression ≥ |1| at any time point. ACO activity was measured as a proxy for ethylene production in wounded and control roots and expressed per mg protein. SAMS, S-adenosyl-L-methionine synthetase; ACS, 1-aminocyclopropane-1-carboxylic acid synthase; ACO, 1-aminocyclopropane-1-carboxylic acid oxidase; ETR, ethylene receptor; CTR1, constitutive triple response 1; EIN, ethylene insensitive; ERF, ethylene response factor. For all analyses, n =4. Error bars are SE of the mean.

To better understand the role of ethylene-responsive transcription factors and ethylene signaling in wounded roots, wound effects on the transcription of genes involved in ethylene biosynthesis were analyzed. Four ethylene biosynthetic genes, including genes for S-adenosyl-L-methionine synthetase (SAMS) and 1-aminocyclopropane-1-carboxylic acid oxidase (ACO) and two genes for 1-aminocyclopropane-1-carboxylic acid synthase (ACS) were significantly upregulated in wounded roots (**Figure 4A**). Upregulation of gene expression was evident within 2 h after wounding for ACS and ACO genes and within 8 h for SAMS. Expression of SAMS, ACS_1, and ACO increased progressively with time after wounding, and by 24 h these genes exhibited log_2_ fold increases of 4.0, 5.6, and 7.9, respectively, or the equivalent of 16-, 50-, and 240-fold elevations in gene expression in wounded roots relative to controls. ACS_2 expression, in contrast, exhibited transient upregulation between 2 and 12 h after injury. The strong upregulation of ACO transcript levels was reflected in elevated ACO enzymatic activity in wounded roots relative to controls (**Figure 4C**) with ACO activity highly correlated (r = 0.912) to ACO gene expression. ACO activity generally declined in unwounded roots after harvest, but increased in wounded roots during the first 8 h after wounding and remained at elevated levels for the remaining 16 h of the experiment. As a result, ACO activity was 1.6 to 2.2-fold greater in wounded roots in the 8 to 24 h after wounding. In addition to its high correlation with ACO gene expression, ACO activity was significantly correlated with SAMS gene expression (r = 0.705).

Genes involved in ethylene signal transduction were also elevated in wounded sugarbeet roots, including genes for ethylene receptors (ETR_1 and ETR_2), constitutive triple response 1 (CTR1), ethylene insensitive (EIN)2, and ethylene-responsive transcription factors (ERF) 1/2 (**Figure 4B**). The most highly upregulated of these genes encoded for ERF1/2_1, EIN2, ERF1/2_2 and ETR_2 which exhibited log_2_ fold expression changes of 6.8, 6.1, 4.4, and 3.8, respectively, by 24 h after wounding, equivalent to 110-, 70-, 21-, and 14-fold changes in expression. The increase in expression for ethylene signal transduction genes (**Figure 4B**) lagged increases in expression for ethylene biosynthetic genes (**Figure 4A**). Upregulation was first evident at 4 h post-wounding for ETR_1 and ETR_2, and 8 h or more for CTR1, EIN2, ERF1/2_1, and ERF1/2_2. EIN3 was unique as the only ethylene signaling pathway gene that was downregulated by wounding. However, downregulation of EIN3 expression occurred only in root samples collected 8 h after wounding; EIN3 expression was unaltered at all other sampling time points. EIN2 and EIN3 were also notable for their significant correlation with ACO activity, with activity positively correlated with EIN2 (r = 0.747) and negatively correlated with EIN3 (r = -0.703). ACO activity was also highly correlated with six AP2-EREBP transcription factors (LOC104898899, r = 0.895; LOC104894972, r = 0.838; LOC104895475, r = 0.772; LOC104902404, r = 0.772; LOC104896121, r = 0.747; LOC104892796, r = 0.744).

### Jasmonic acid biosynthesis and signal transduction

**Figure 5:**
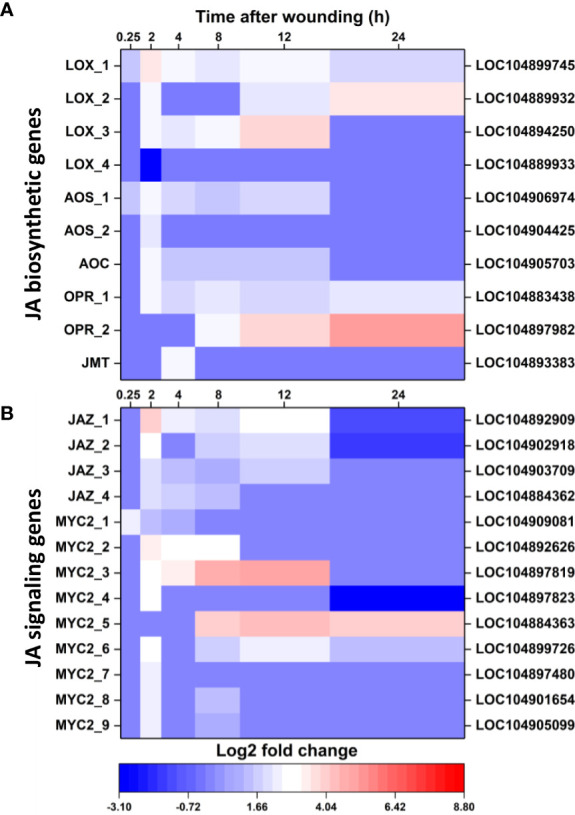
Changes in differential expression with respect to time after wounding for genes involved in jasmonic acid (JA) biosynthesis **(A)** and JA signaling **(B)**. Heat maps display the log_2_ fold change in gene expression between wounded and control roots at 0.25, 2, 4, 8, 12 and 24 h after wounding for all pathway genes that exhibited a log_2_ fold change in expression ≥ |1| at any time point. LOX, lipoxygenase; AOS, allene oxide synthase; AOC, allene oxide cyclase; OPR, 12-oxophytodienoic acid reductase; JMT, jasmonate O-methyltransferase; JAZ, jasmonate ZIM domain-containing protein. For all analyses, n = 4.

Wounding altered the expression of genes involved in JA biosynthesis and the JA signal transduction pathway. Ten JA biosynthetic genes were differentially expressed in wounded roots relative to controls (**Figure 5A**). Nine of these DEGs were upregulated in response to wounding and included three lipoxygenase (LOX) genes, two allene oxide synthase (AOS) genes, one allene oxide cyclase gene, two 12-oxophytodienoic acid reductase (OPR) genes, and one jasmonate O-methyltransferase gene (JMT). The majority of JA biosynthetic DEGs were transiently altered in expression between 2 and 12 h after wounding and, by 24 h, only two LOX genes and two OPR genes were upregulated in wounded roots. Although the number of JA biosynthetic DEGS (**Figure 5A**) exceeded the number of ethylene biosynthetic DEGs (**Figure 4A**), the level of upregulation of JA biosynthetic DEGs was notably lower than that of the ethylene biosynthesis DEGs. Of JA biosynthetic DEGs, OPR_2 was the most highly upregulated with a log_2_ fold change in expression of 4.9 after 24 h.

Wounding also altered expression of 13 genes involved in JA signaling, with all genes upregulated a minimum of one time point in the 24 h after wounding (**Figure 5B**). JA signal transduction DEGs included four jasmonate ZIM domain-containing protein (JAZ) genes and nine MYC2 genes (**Figure 5B**). These genes were generally upregulated transiently between 2 and 12 h after wounding, although two genes, MYC2_5 and MYC2_6 remained upregulated at 24 h. Overall, JA signal transduction DEGs (**Figure 5B**) were upregulated to a lesser extent than ethylene signal transduction DEGs (**Figure 4B**). Greatest upregulation among JA signaling DEGs was observed for MYC2_3 at 12 h, which had a 5.1 log_2_ fold increase, equivalent to a 34-fold increase, in expression.

### Phenylpropanoid biosynthetic pathway

**Figure 6:**
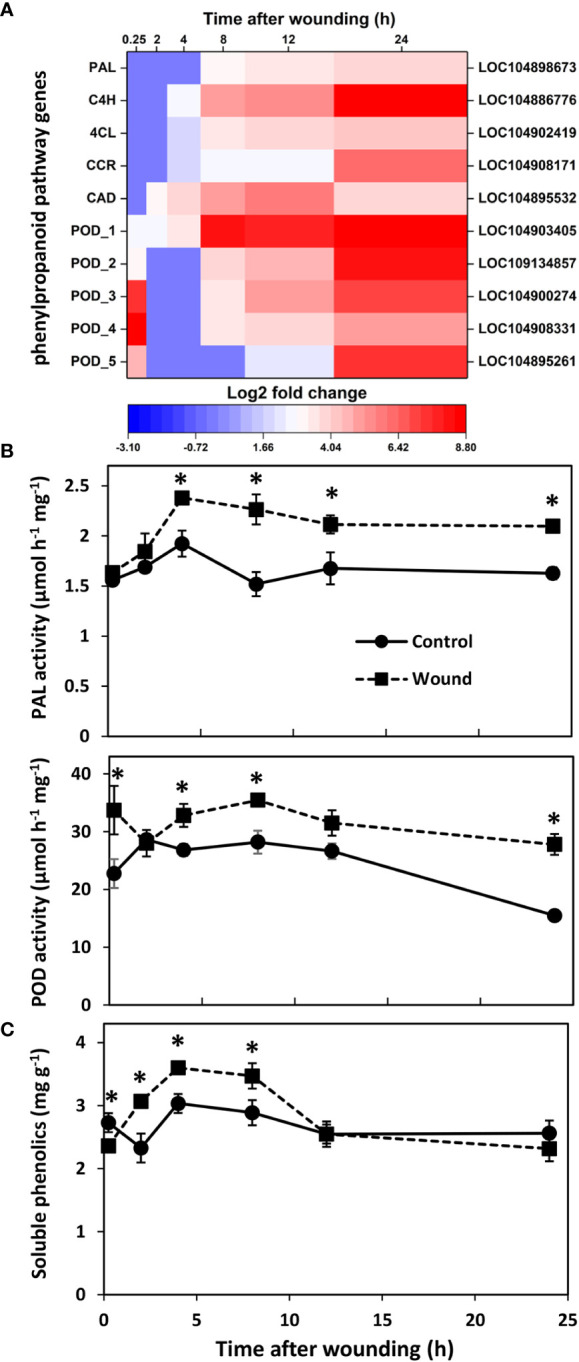
Changes in **(A)** differential expression of genes involved in the production or polymerization of phenolic substrates for suberin and lignin formation, **(B)** PAL and POD enzyme activities, and **(C)** soluble phenolics concentration with respect to time after wounding. Heat map displays the log_2_ fold change in gene expression between wounded and control roots at 0.25, 2, 4, 8, 12 and 24 h after wounding for all pathway genes that exhibited a log_2_ fold change in expression ≥ |1| at any time point after wounding. Enzyme activities are expressed per mg protein; soluble phenolics concentration is expressed as mg gallic acid per g dry weight. PAL, phenylalanine ammonia-lyase; C4H, trans-cinnamate 4-monooxygenase; 4CL, 4-coumarate:coenzyme A (CoA) ligase; CCR, cinnamoyl-CoA reductase; CAD, cinnamyl alcohol dehydrogenase; POD, peroxidase. For all analyses, n = 4. Error bars are SE of the mean. * denotes time points for which values for wounded and control roots differed significantly (p ≤ 0.05).

Wound effects on the transcription of genes involved in the production and polymerization of the phenolic substrates for suberin and lignin formation were also determined due to the importance of lignin and suberin biosynthesis in healing plant injuries and the high level of enrichment of the phenylpropanoid biosynthetic pathway in injured roots (**Figure 2**). Five genes that contribute to the production of the phenolic precursors of lignin and suberin were highly upregulated after wounding (**Figure 6A**). These included genes encoding phenylalanine ammonia-lyase (PAL), trans-cinnamate 4-monooxygenase (C4H), 4-coumarate:coenzyme A (CoA) ligase (4CL), cinnamoyl-CoA reductase (CCR), and cinnamyl alcohol dehydrogenase (CAD). Upregulated expression was evident within the first 2 h after wounding for CAD, within 4 h for C4H, 4CL, and CCR, and within 8 h for PAL, with the expression of all five genes generally increasing with time after wounding. By 24 h, these five genes were upregulated 3.8 to 8.7 log_2_ fold, equivalent to 15 to 400-fold, in wounded roots relative to controls. Wounding also increased expression of five peroxidase genes. These genes that encode the enzyme that initiates the polymerization of phenolic compounds into lignin and suberin, were upregulated in wounded roots within 15 minutes after injury. Although expression of four of the five POD genes transiently declined within 2 h after wounding, all five POD genes were highly upregulated by 24 h with log_2_ fold changes in expression of 4.9 to 8.8, equivalent to 30 to 450-fold greater expression in wounded roots than in controls.

PAL and POD enzyme activities reflected the transcriptional upregulation that was observed in wounded roots (**Figure 6B**). PAL activity displayed high correlation with the expression of PAL, C4H, 4CL, and CAD genes (r = 0.724, 0.849, 0.731, and 0.743, respectively) as well as with two genes of the AP2-EREBP family of transcription factors (LOC104895475, r = 0.927; LOC104894972, r = 0.891). In contrast, POD activity was not significantly correlated with the expression of any individual POD gene but correlated significantly with the expression of three WRKY transcription factors (LOC104900259, r = 0.729; LOC104888587, r = 0.709; LOC104906019, r = 0.708). On average, PAL activity was elevated 31% in wounded roots from 4 to 24 h after wounding. POD activity was significantly elevated in wounded roots within 15 minutes after injury and was present at levels that were 80% greater in wounded roots relative to controls after 24 h. Soluble phenolic compounds that are products of the phenylpropanoid pathway were present at higher concentrations in wounded roots between 2 and 8 h after injury (**Figure 6C**). In wounded roots, soluble phenolics concentration was elevated 50% between 0.25 to 4 h but was comparable to controls at 12 and 24 h after wounding.

## Discussion

### Wounding causes widespread changes in transcription and signaling

Wounding of freshly harvested sugarbeet roots led to rapid and widespread changes in gene expression in the first 24 h after injury. Wounding caused more than 700 genes to be differentially expressed within 15 minutes of injury. The number of DEGs increased logarithmically during the first 8 h after injury, and within 24 h, nearly 5000 genes were differentially expressed-equivalent to 21% of the number of expressed genes that were detected in roots. The proportion of expressed genes that were altered by wounding was significantly greater in sugarbeet root than has been reported for plant leaves. In leaf tissue of both *Arabidopsis* and chickpea, wounding altered expression of approximately 8% of analyzed or identified genes (Cheong et al., 2002; Pandey et al., 2017).

DEGs in wounded sugarbeet roots contributed to a broad range of biological and molecular processes as described by GO terms and KEGG orthologies. The effect of wounding on gene expression, therefore, was vast and affected cellular functions and pathways well beyond those needed for cell repair. The most highly populated GO terms related to metabolism at the cellular and organismal level, indicating a major reallocation of metabolism both locally and systemically. The mapping of DEGs to KEGG pathways further established that primary as well as secondary metabolic pathways were altered by wounding.

In line with a major reallocation of metabolism, an abundance of DEGs were involved in signaling. Plant hormone signal transduction and MAPK signaling pathways were the most highly enriched pathways in wounded roots within the first 24 h after injury. The importance of plant hormone signaling, especially signaling pathways involving jasmonic acid and ethylene, is well established in other plant wounded tissues (Koo et al., 2009; Savatin et al., 2014). Similarly, MAPK kinases have been implicated in wound and hormonal signaling in other plant species (Sinha et al., 2011). In the present study, 514 transcription factors belonging to 50 TF families were differentially expressed. These TF genes accounted for nearly 6% of all DEGs. The greatest number of TF DEGs belonged to the AP2-EREBP, MYB, bHLH, WRKY and NAC transcription factor families. AP2-EREBP TFs participate in ethylene signaling and activate both defense and wound repair mechanisms (Gutterson and Reuber, 2004; Heyman et al., 2018). MYB and NAC TFs are known contributors to the regulation of secondary cell wall formation and the phenylpropanoid pathway (Bomal et al., 2008; Nakano et al., 2015); bHLH TFs are known to activate plant defense mechanisms and secondary metabolite biosynthesis (Schwartz et al., 2017; Wang et al., 2020) and WRKY TFs commonly induce defense genes and modulate JA and salicylic acid accumulation (Rushton et al., 2010). Differential expression of members of these TF families provides evidence for the importance of hormonal signaling as well as activation of plant defense mechanisms, cell wall repair, and secondary metabolism in wounded sugarbeet roots. Wound-induced alterations in expression of these TF families is not unique to sugarbeet root and have been reported in other plant species and organs (Pandey et al., 2017; Dombrowski et al., 2020; Lim et al., 2021).

### Wounding alters expression of ethylene and jasmonic acid pathways

Ethylene and jasmonic acid biosynthesis are well-established wound responses in plants (O’Donnell et al., 1996; Li et al., 2001; Vega-Muñoz et al., 2020). Once synthesized, these hormones trigger signaling cascades that induce cell repair and plant defense mechanisms that subsequently seal off wound sites and protect against opportunistic pathogens (Onkokesung et al., 2010; Savatin et al., 2014; Heyman et al., 2018). In wounded sugarbeet roots, a total of 34 genes involved in ethylene and JA biosynthesis and signal transduction were differentially expressed in the 24 h after injury, with all but two of these genes upregulated. Ethylene and JA, therefore, are likely to have significant roles in initiating sugarbeet root responses to injury.

DEGs involved in ethylene biosynthesis and signal transduction were predominantly upregulated in wounded roots from 8 to 24 h after injury, with greatest induction occurring after 24 h. Genes encoding all necessary enzymes for the biosynthesis of ethylene from methionine were significantly upregulated in wounded roots, with genes for ACS and ACO, the two rate-limiting enzymes for ethylene production (Wang et al., 2002; Pattyn et al., 2021), upregulated by as much as 60- and 240-fold, respectively. Whether ACS or ACO restricts ethylene production rate varies between plant species, organ, or environment. In general, ACS limits ethylene biosynthesis under non-stressed conditions, while ACO is limiting in some non-leaf and abiotically stressed tissues (Houben and Van de Poel, 2019; Pattyn et al., 2021). Genes involved in ethylene signaling were also upregulated, including genes for ethylene receptors, EIN2, and ethylene responsive factors that interact with gene promoters to invoke ethylene responses. ERF1/2 and EIN2 were the most highly upregulated ethylene signaling genes in wounded sugarbeet roots and were upregulated after 24 h by as much as 110- and 70-fold, respectively. ERF1/2 genes have been shown to induce the transcription of plant defense genes (Lorenzo et al., 2003; Yang et al., 2021), while EIN2 is a central positive regulator of ethylene responses that acts upstream of ERFs in the ethylene signaling pathway (Alonso et al., 1999). Other genes in the canonical ethylene signaling pathway, such as CTR1, a negative regulator of ethylene signaling, and EIN3, a positive regulator that operates downstream of EIN2, were largely unaffected by wounding. Overall, the induction of ethylene biosynthesis DEGs preceded induction of ethylene signaling DEGs, with biosynthesis-related DEGs upregulated as early as 2 h after injury and signaling-related DEGs first upregulated between 4 to 8 h after injury. Results of the current study are consistent with the upregulation of genes involved in ethylene biosynthesis and signaling in response to wounding that has been reported in other plant species and organs (Kato et al., 2000; Pandey et al., 2017; Dombrowski et al., 2020; Lim et al., 2021). Results are also consistent with an earlier study that found that wounding accelerated ethylene production and likely induced ethylene receptor production in harvested sugarbeet roots (Fugate et al., 2010).

Upregulated DEGs involved in jasmonate biosynthesis included genes for the first four of eight enzymes needed to synthesize JA from α-linolenic acid as well as an enzyme that converts JA to its methyl ester. JA signaling DEGs were also upregulated including genes for JAZ, a repressor of JA signaling, and MYC2, a transcription factor involved in activating JA-responsive genes (Ruan et al., 2019). Most JA-related DEGs were transiently upregulated between 2 and 12 h after wounding, with three of the 23 JA-related DEGs induced within 15 minutes. By 24 h, however, transcription levels of JA-related DEGs had mostly declined to levels equal or below those of control roots. In general, the expression of JA-related DEGs shadowed that of WRKY TF DEGs. Although the role of WRKY TFs in JA-related gene expression in sugarbeet root is not known, WRKY TFs are known to modulate JA concentrations in other plant species (Rushton et al., 2010; Zhao et al., 2020). The extent of upregulation of JA-related DEGS varied from 2 to 34-fold during the 24 h after injury, with 25% of DEGs induced by 10-fold or more at one or more time points. In contrast, ethylene-related DEGs were upregulated 2 to 110-fold with 75% of DEGs induced 10-fold or more at some time in the 24 h after wounding. Upregulation of JA-related genes, therefore, preceded ethylene-related DEG upregulation, reached maximum induction prior to ethylene-related genes, and generally returned to control levels while ethylene-related transcript levels continued to increase. Moreover, JA-related DEGS were upregulated to much lower levels than ethylene-related DEGS.

Although both ethylene and JA biosynthetic and signaling genes were upregulated after root injury, evidence suggests that wound signaling *via* ethylene pathways predominates over jasmonate pathways in harvested sugarbeet roots. Throughout the 24 h after wounding, ethylene-related genes were induced to greater levels and longer durations than JA-related genes. Additionally, four JAZ genes were upregulated between 2 and 12 h, potentially increasing the concentration of proteins that repress JA action (Koo et al., 2009; Ruan et al., 2019). Because ethylene inhibits JA biosynthesis and signaling in other plant species (Xiong et al., 2017; Munemasa et al., 2019), the large upregulation of ethylene-related genes potentially suppressed JA signaling in wounded sugarbeet roots. A limited JA response in wounded sugarbeets, therefore, is proposed based on the results of this study despite JA’s central role in wound signaling in most plant species and organs (Marhavý et al., 2019). Like the current study, JA signaling was found to be of minimal importance in wounded roots of *Arabidopsis* (Savatin et al., 2014).

### Wounding induces phenylpropanoid pathway and peroxidase genes

Wounding induced the transcription of five phenylpropanoid pathway enzymes that are central to the synthesis of the phenolic compounds that are substrates for suberin, lignin and flavonoid biosynthesis and are needed to seal off wound sites, repair cell damage, and defend against pathogens (Zhang and Liu, 2015). Upregulated phenylpropanoid pathway DEGs included genes for PAL, the main regulator of flux into the pathway, and C4H and 4CL, which together with PAL catalyze the first three pathway reactions and are required to produce all pathway products (Bates et al., 1994; Vogt, 2010). Also upregulated were genes for CCR and CAD, essential enzymes in the synthesis of monolignols which are utilized in suberin and lignin synthesis (Vogt, 2010). Phenylpropanoid-related DEGs were upregulated by as much as 15- to 416-fold relative to unwounded roots. In general, upregulation of phenylpropanoid-related DEGs began 4 h after injury and increased progressively during the 24 h after injury. Although ethylene and JA have been implicated in the upregulation of the phenylpropanoid pathway in other plant species (Ecker and Davis, 1987; Chen et al., 2006), the involvement of these hormones in regulating the phenylpropanoid pathway in wounded sugarbeet roots is unknown. However, similarities in expression patterns between phenylpropanoid pathway and ethylene-related DEGs, but not JA-related DEGs, were apparent, and a high level of correlation was found between PAL activity and the expression of two ethylene-responsive transcription factors. Like the present study, the upregulation of phenylpropanoid pathway genes in the 24 h after wounding is reported in other plant species and organs (Reymond et al., 2000; Becerra-Moreno et al., 2015; Pandey et al., 2017; Si et al., 2020).

Peroxidase genes were also highly upregulated, with their expression induced by as much as 170- to 450-fold relative to unwounded control roots. POD DEGs predominantly displayed a biphasic response to wounding, with an initial transient upregulation in the first 15 minutes that subsided by 2 h and a second progressive upregulation in expression between 8 and 24 h after injury. Peroxidases are multifunctional enzymes that oxidize a variety of substrates to generate hydrogen peroxide and reactive oxygen species (ROS) and function in the initiation of suberin and lignin polymerization reactions and the control of cellular hydrogen peroxide concentrations (Passardi et al., 2005). With such reactivity, PODs play key roles in the production and regulation of the oxidative burst that occurs within minutes after injury as well as later cell wall polymerization reactions that generate suberin and lignin barriers at wound sites and fortify cell walls (Minibayeva et al., 2015; Prasad et al., 2020). While the role of PODs in wounded sugarbeet roots has not been examined, the rapid early and later sustained induction of POD DEGs suggest that PODs are involved in both an early oxidative burst and later polymerization and cross-linking reactions in injured roots.

### Activities of key enzymes and soluble phenolics concentration mirror transcriptional changes

Enzymatic activities of ACO, PAL and POD, three rate-limiting enzymes with highly upregulated transcript levels, mirrored transcriptional changes in wounded sugarbeet roots. Changes in enzymatic activities, however, were moderate reflections of the dramatic changes that occurred at the transcriptional level. The cause for differences in the intensity of changes in transcript and activity levels is unknown. However, the three enzymes that were assayed are products of large gene families of which only one or a few family members were altered in expression due to wounding. Activity changes arising from DEGs, therefore, may be tempered by background activity arising from the transcription and translation of non-differentially expressed gene family members. Alternatively, differences between changes in transcript levels and enzyme activity may reflect post-transcriptional regulation of ACO, PAL and POD gene expression. Similar dampening of transcriptional changes that was reflected in smaller alterations in enzyme activities has been reported previously (Gibon et al., 2004; Gibon et al., 2006).

The concentration of soluble phenolic compounds is dependent on both their rate of biosynthesis *via* the phenylpropanoid pathway and their rate of usage, most notably as substrates for suberin and lignin biosynthesis. In wounded sugarbeet roots, the concentration of soluble phenolics was elevated between 2 and 8 h after injury but unaltered at 12 and 24 h. The transient increase in soluble phenolic compounds is speculated to reflect both the upregulation of the phenylpropanoid pathway increasing phenolic compound biosynthesis, and the upregulation of POD genes that were responsible for their utilization, most likely as substrates for suberin biosynthesis. While phenolic compounds could also serve as substrates for lignin biosynthesis, their incorporation into this polymer was unlikely since lignin formation in wounded sugarbeet roots is not apparent during the first week after injury (Ibrahim et al., 2001; Fugate et al., 2016).

## Conclusion

Wounding of sugarbeet roots caused large transcriptional changes in the 24 h following injury indicating a widespread reallocation in root metabolism. While transcript levels of differentially expressed genes were involved in a vast array of molecular and cellular functions, an abundance of transcription factor genes and genes involved in ethylene and jasmonate signaling was noted. The upregulation of genes involved in ethylene and JA biosynthesis and signaling suggest that both of these hormones play a role in sugarbeet root wound responses. Signaling *via* ethylene, however, was likely of greater importance than JA since ethylene-related genes were upregulated to greater levels and longer durations than JA-related genes and repressors of JA signaling were also induced. Genes involved in the biosynthesis and polymerization of phenolic compounds were also highly upregulated in the 24 h following injury. The biosynthesis of monolignols and their polymerization, most likely into suberin, therefore, is likely to be initiated within the first 24 h after injury in sugarbeet roots.

## Data availability statement

The datasets presented in this study can be found in online repositories. The names of the repository/repositories and accession number(s) can be found below: https://www.ncbi.nlm.nih.gov/, BioProject PRJNA762331.

## Author contributions

FF and KF conceived the study, analyzed data, and wrote the manuscript. FF, MD, and AL performed or assisted with experiments. MK contributed to the supervision of research. All authors contributed to the article and approved the submitted version.
